# Wafer Center Alignment System of Transfer Robot Based on Reduced Number of Sensors

**DOI:** 10.3390/s22218521

**Published:** 2022-11-05

**Authors:** Hyungjong Kim

**Affiliations:** Korea Institute of Industrial Technology, Ansan 426-171, Korea; hyungjong@gmail.com; Tel.: +82-31-8040-6384; Fax: +82-31-8040-6390

**Keywords:** semiconductor wafer, wafer center alignment, three optical sensors, wafer transfer robot

## Abstract

This brief presents a wafer alignment algorithm with reduced sensor number that obtains the relative distance of the wafer center and the robot hand. By ‘reduced number’, in spite of smaller number of sensors than the conventional method, we mean an improved method which achieves the similar results to pre-existing algorithms. Indeed, it can be designed with only three sensor data, less than four sensors of the conventional algorithm. Thus, some advantages of the proposed alignment algorithm include that it can be designed with low cost and less computing power. The proposed alignment algorithm is applied to a transfer robot for coater/developer system in semiconductor processing for verifying the performance of the method. The performance of the proposed method is validated by both simulation and experimental results.

## 1. Introduction

The alignment problem of wafer center is a significant and critical topic in semiconductor manufacturing processing because it affects coating performance, chemical edge bead removal (EBR), and the backside rinse steps for lithographic applications [1]. Its effect increases when the semiconductor process enters the era of deep submicron meter (below 20 nm), e.g., EUV process. In order to solve the alignment problem, there are several approaches [2,3,4,5,6,7,8,9,10,11,12,13,14]. The most typical device is wafer pre-alignment system (which is called pre-aligner) that consists of rotation mechanics and optical vision sensor [6]. Although it can accurately detect the position of geometric center and notch direction, the system requires a rotating mechanism in order to find the information of maximum eccentricity and the notch, and then the motion process causes a delay in the processing. Instead of the rotational mechanism, a method using the straight motion of the robot hand holding the wafer was studied in [15,16]. The method, called AWC (automatic wafer centering), uses several thru-beam sensors to calculate the center of the wafer from the distance across it. Although this method is widely used in vacuum robots, the method also requires additional motion to obtain sensing information. To solve this, the authors of [17] have proposed an automatic alignment system that can detect the wafer center without the rotating mechanism. It consists of robot hand for wafer transfer and four vision sensors to obtain the wafer position information. Thus, without the additional aligner, it can align the wafer center whenever the transfer robot has the wafer on robot hand.

In this paper, we mainly focus on the case where the number of the sensors is one less than the previous studies. For this, the proposed algorithm is designed with only three sensor data, less than four sensors of the conventional method. Thus, some advantages of the proposed alignment algorithm include that it can be designed with low cost and less computing power. For the constructive design of the proposed method, we suggest some assumptions and provide mathematical proofs under them. In order to verify the performance, it is finally applied to a transfer robot for coater/developer system in semiconductor process.

The paper is organized as follows: in Section 2, an algorithm for finding the wafer center is presented. For this, we introduce some assumptions and lemmas for theoretical support (see Section 2.1). Moreover, Section 2.2 provides an algorithm to avoid a wafer notch. In Section 3, the proposed algorithm is experimentally tested for the transfer robot of coater/developer system. Finally, Conclusions are found in Section 4.

*Notation*: Cartesian plane with the familiar x- and y-axes is considered. The vector from A to B is denoted by $\vec{AB}$; the point A is called its tail and the point B is called its head. Then, the set of all points in the place corresponds to the set of all vectors whose tails are at the origin O. For example, the point A=(1,2) and we write the vector $\vec{a}=\vec{OA}={\left[\begin{array}{cc} 1 & 2 \end{array}\right]}^{⊤}$ using square brackets where $\cdot^{⊤}$ implies the matrix transpose [18]. In this paper, all points are represented as a vector with the origin O as tail.

## 2. Main Results

**Figure 1:**
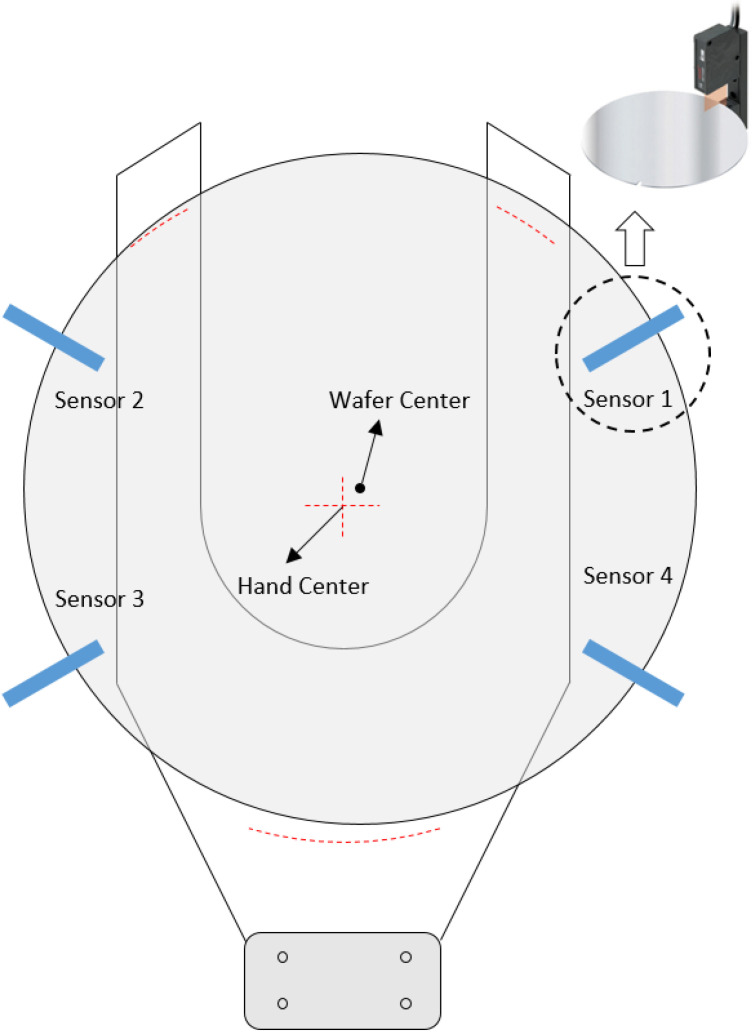
Wafer center alignment system with four sensors.

**Figure 2:**
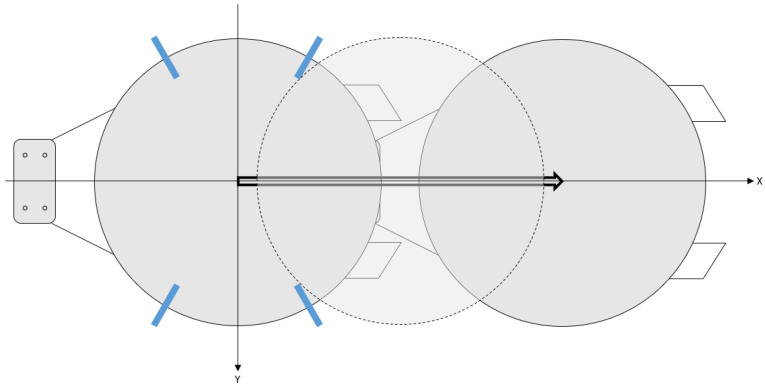
Motion of robot hand holding the wafer (with four sensors).

Here, a detection method of wafer center is discussed that can be designed with only three sensor data as mentioned in the previous section. As is well known in the literature, it is possible to derive the wafer center from the circumcenter of a triangle because the shape of the wafer is a circle and the three sensor information are known. However, it may not be possible to determine the exact center due to the notch on the edge of the wafer, as shown in Figure 1. To solve this problem, the proposed method is composed of two parts. The first part is to obtain the wafer center from three sensor data and the known wafer radius *r*. In the second part, with the movement of the robot hand as shown in Figure 2, we introduce an algorithm for avoiding the notch cope with abnormal cases.

**Figure 3:**
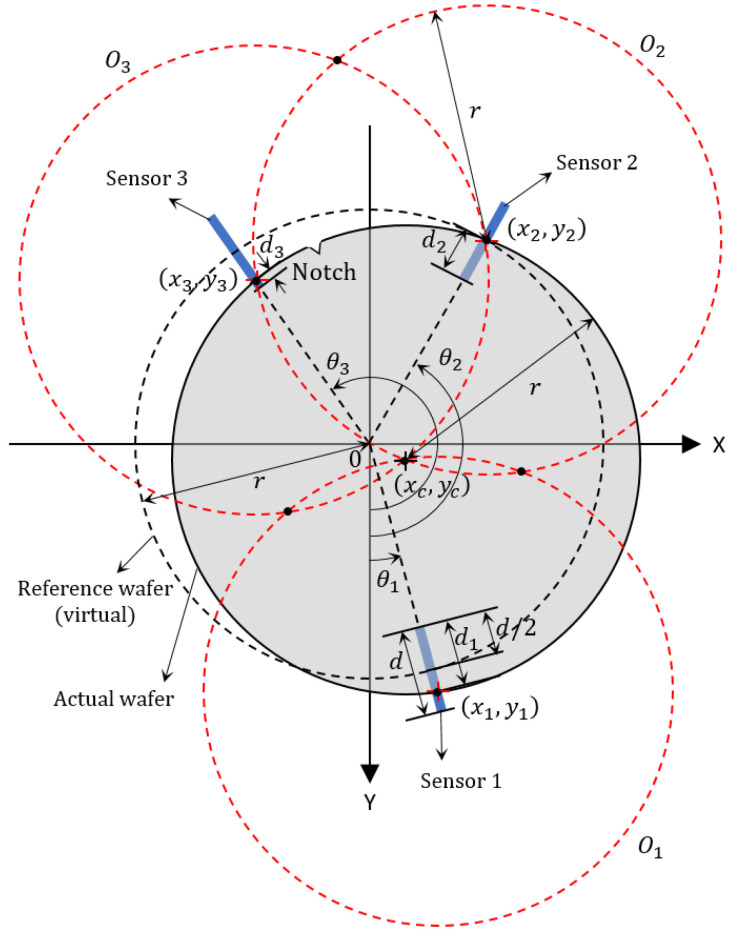
Configuration of alignment system with three sensors.

Now we consider the case of three sensors, and then its configuration with the installed sensors is shown in Figure 3. It is supposed that the measurable sensor range *d* is known. Additionally, the angles of installed sensors $\theta_{1},\theta_{2}$, and $\theta_{3}$ with respect to the *Y-axis* are known distinct positive number and it is assumed that $0\leq \theta_{1}<\theta_{2}<\theta_{3}<360^{\circ }$. In order to obtain the actual wafer center ${\left[\begin{array}{cc} x_{c} & y_{c} \end{array}\right]}^{⊤}$, we assume the followings.

**Assumption 1.**  *The range d is less than the wafer diameter 2r, i.e., $r-\frac{d}{2}>0$. For the sensor number i=1,2,3, the sensing range (bold blue line in Figure 3) is a connected compact set $S_{i}$ as follows:*

$$S_{i}:=\left\{\left(r+\lambda d\right){\left[\begin{array}{cc} \sin(\theta_{i}) & \cos(\theta_{i}) \end{array}\right]}^{⊤}\in R^{2}|-\frac{1}{2}\leq \lambda \leq \frac{1}{2}\right\}.$$

**Assumption 2.**  *The actual wafer center is limited as follows:*

$$\begin{array}{c} {\left[\begin{array}{cc} x_{c} & y_{c} \end{array}\right]}^{⊤}\in \left\{{\left[\begin{array}{cc} x & y \end{array}\right]}^{⊤}\in R^{2}|\sqrt{x^{2}+y^{2}}\leq \frac{d}{2}\right\}=:W. \end{array}$$

**Assumption 3.**  *If $90^{\circ }\leq \left|\theta_{i}-\theta_{j}\right|\leq 270^{\circ }$ for any $i,j\in \left\{1,2,3|i\neq j\right\}$, $|\cos\left(\theta_{i}-\theta_{j}\right)|<2{\left(\frac{r}{r+d/2}\right)}^{2}-1$. Otherwise, $d<2(\sqrt{2}-1)r$.*

Here, Assumption 1 and 2 are acceptable conditions for the sensors and the wafer location to detect the wafer edge. Assumption 3 is required in order to identify the wafer center (to be discussed).

### 2.1. Center Calculation Algorithm

In this section, we propose an algorithm to find wafer center from sensor values. As shown in Figure 3, it follows that three points ${\left[\begin{array}{cc} x_{i} & y_{i} \end{array}\right]}^{⊤}$ for i=1,2,3 at which the wafer edge is detected by the sensors are given by

(1) $$\begin{array}{c} \begin{array}{cc} x_{i} & =\left(r-d/2+d_{i}\right)\sin\theta_{i},\\ y_{i} & =\left(r-d/2+d_{i}\right)\cos\theta_{i} \end{array} \end{array}$$

where $d_{i}$’s are measurable sensor values. The existence of ${\left[\begin{array}{cc} x_{i} & y_{i} \end{array}\right]}^{⊤}$ is proved in the following lemma.

**Lemma 1.**  *For any ${\left[\begin{array}{cc} x_{c} & y_{c} \end{array}\right]}^{⊤}\in W$, there exists a point ${\left[\begin{array}{cc} x_{i} & y_{i} \end{array}\right]}^{⊤}\in S_{i}$, such that ${\left(x_{i}-x_{c}\right)}^{2}+{\left(y_{i}-y_{c}\right)}^{2}=r^{2}$ for i=1,2,3.*

**Proof.**  Equation (1) can be represented by

(2) $$\begin{array}{c} \left[\begin{array}{c} x_{i}\\ y_{i} \end{array}\right]=\left(r+\lambda_{i}d\right)\left[\begin{array}{c} \sin(\theta_{i})\\ \cos(\theta_{i}) \end{array}\right],\;\lambda_{i}=\frac{d_{i}}{d}-\frac{1}{2}. \end{array}$$

Here, it follows from Assumption 1 that ${\left[\begin{array}{cc} x_{i} & y_{i} \end{array}\right]}^{⊤}\in S_{i}$ because $0\leq d_{i}\leq d$. Now, let

$$\begin{array}{c} \left[\begin{array}{c} {\bar{x}}_{i}\\ {\bar{y}}_{i} \end{array}\right]:=R\left(\theta_{i}\right)\left[\begin{array}{c} x_{i}\\ y_{i} \end{array}\right]\;\mathrm{and}\;\left[\begin{array}{c} {\bar{x}}_{c,i}\\ {\bar{y}}_{c,i} \end{array}\right]:=R\left(\theta_{i}\right)\left[\begin{array}{c} x_{c}\\ y_{c} \end{array}\right] \end{array}$$

where $R(\theta_{i})$ is a rotation matrix as follows:

(3) $$\begin{array}{c} R\left(\theta_{i}\right):=\left[\begin{array}{cc} \cos\theta_{i} & -\sin\theta_{i}\\ \sin\theta_{i} & \cos\theta_{i} \end{array}\right]. \end{array}$$

Then, by the Equations (2) and (3), we obtain that

$$\begin{array}{c} \left[\begin{array}{c} {\bar{x}}_{i}\\ {\bar{y}}_{i} \end{array}\right]=R\left(\theta_{i}\right)\left[\begin{array}{c} x_{i}\\ y_{i} \end{array}\right]=\left[\begin{array}{c} 0\\ r+\lambda_{i}d \end{array}\right]. \end{array}$$

It is easy to show that ${\left[\begin{array}{cc} {\bar{x}}_{i} & {\bar{y}}_{i} \end{array}\right]}^{⊤}$ with $\lambda_{i}=\frac{1}{d}\left(\sqrt{r^{2}-{\bar{x}}_{c,i}^{2}}-r+{\bar{y}}_{c,i}\right)$ satisfies the equation ${\left({\bar{x}}_{i}-{\bar{x}}_{c,i}\right)}^{2}+{\left({\bar{y}}_{i}-{\bar{y}}_{c,i}\right)}^{2}=r^{2}$. Moreover, it can be shown that $-\frac{1}{2}\leq \lambda_{i}\leq \frac{1}{2}$ as follows:

1. Since ${\left[\begin{array}{cc} {\bar{x}}_{c,i} & {\bar{y}}_{c,i} \end{array}\right]}^{⊤}\in W$, it follows from Assumption 2 that $-\frac{d}{2}\leq {\bar{y}}_{c,i}\leq \frac{d}{2}$. Then, we can obtain
  (4) $$\begin{array}{cc} \lambda_{i} & =\frac{1}{d}\left(\sqrt{r^{2}-{\bar{x}}_{c,i}^{2}}-r+{\bar{y}}_{c,i}\right)\\ \; & \leq \frac{1}{d}\left(\sqrt{r^{2}}-r+{\bar{y}}_{c,i}\right)=\frac{1}{d}{\bar{y}}_{c,i}\leq \frac{1}{2}. \end{array}$$
2. Next, we have
  $$\begin{array}{cc} \lambda_{i} & =\frac{1}{d}\left(\sqrt{r^{2}-{\bar{x}}_{c,i}^{2}}-r+{\bar{y}}_{c,i}\right)\\ \; & =\sqrt{{\left(\frac{r}{d}\right)}^{2}-{\left(\frac{{\bar{x}}_{c,i}}{d}\right)}^{2}}-\frac{r}{d}+\frac{{\bar{y}}_{c,i}}{d}\\ \; & \geq \sqrt{{\left(\frac{r}{d}\right)}^{2}-\frac{1}{4}+{\left(\frac{{\bar{y}}_{c,i}}{d}\right)}^{2}}-\frac{r}{d}+\frac{{\bar{y}}_{c,i}}{d}. \end{array}$$
  Define $\eta :=\frac{{\bar{y}}_{c,i}}{d}$. Then, $-\frac{1}{2}\leq \eta \leq \frac{1}{2}$ and
  $$\begin{array}{c} f\left(\eta \right):=\sqrt{{\left(\frac{r}{d}\right)}^{2}-\frac{1}{4}+\eta^{2}}-\frac{r}{d}+\eta \end{array}$$
  is a monotonically increasing function on η ([19], [page 95]) because it follows from r−d/2>0 of Assumption 1 that its derivative with respect to η satisfies that
  $$\begin{array}{c} \dot{f}\left(\eta \right)=\eta {\left({\left(\frac{r}{d}\right)}^{2}-\frac{1}{4}+\eta^{2}\right)}^{-\frac{1}{2}}+1>0. \end{array}$$
  This implies that
  (5) $$\begin{array}{c} \lambda \geq f\left(\eta \right)\geq f\left(-\frac{1}{2}\right)=-\frac{1}{2}. \end{array}$$
  Thus, by the Equation (4) and (5), we have
  $$\begin{array}{c} -\frac{1}{2}\leq \lambda =\frac{1}{d}\left(\sqrt{r^{2}-{\bar{x}}_{c,i}^{2}}-r+{\bar{y}}_{c,i}\right)\leq \frac{1}{2}. \end{array}$$

Finally, it follows from the matrix *R* of (3) that

$$\begin{array}{c} {\left({\bar{x}}_{i}-{\bar{x}}_{c,i}\right)}^{2}+{\left({\bar{y}}_{i}-{\bar{x}}_{y,i}\right)}^{2}=r^{2}\;\Rightarrow \;{\left(x_{i}-x_{c}\right)}^{2}+{\left(y_{i}-y_{c}\right)}^{2}=r^{2}. \end{array}$$

Then, for any ${\left[\begin{array}{cc} x_{c} & y_{c} \end{array}\right]}^{⊤}\in W$, $R^{-1}\left(\theta_{i}\right){\left[\begin{array}{cc} 0 & r+\lambda_{i}d \end{array}\right]}^{⊤}\in S_{i}$ with

$\lambda_{i}=\frac{1}{d}\left(\sqrt{r^{2}-{\left(x_{c}\cos\left(\theta_{i}\right)-y_{c}\sin\left(\theta_{i}\right)\right)}^{2}}-r+x_{c}\sin\left(\theta_{i}\right)+y_{c}\cos\left(\theta_{i}\right)\right)$ is the point that satisfies the equation ${\left(x_{i}-x_{c}\right)}^{2}+{\left(y_{i}-y_{c}\right)}^{2}=r^{2}$. The proof is complete since the matrix $R(\theta_{i})$ is invertible [18]. □

Now, from the Equation (1), we can obtain the following equation of the three circles with radius *r* (red dashed circles in Figure 3)

$$\begin{array}{c} O_{i}:=\left\{{\left[\begin{array}{cc} x & y \end{array}\right]}^{⊤}\in R^{2}\;|\;{\left(x-x_{i}\right)}^{2}+{\left(y-y_{i}\right)}^{2}=r^{2}\right\},\;i=1,2,3. \end{array}$$

Then, it follows that we can obtain two intersections for each pair $\left\{O_{1},O_{2}\right\}$, $\left\{O_{1},O_{3}\right\}$, $\left\{O_{2},O_{3}\right\}$ as follows:

(6) $$\begin{array}{cc} O_{1}\cap O_{2} & =\left\{{\left[\begin{array}{cc} x_{1,1} & y_{1,1} \end{array}\right]}^{⊤},{\left[\begin{array}{cc} x_{1,2} & y_{1,2} \end{array}\right]}^{⊤}\right\}, \end{array}$$

(7) $$\begin{array}{cc} O_{1}\cap O_{3} & =\left\{{\left[\begin{array}{cc} x_{2,1} & y_{2,1} \end{array}\right]}^{⊤},{\left[\begin{array}{cc} x_{2,2} & y_{2,2} \end{array}\right]}^{⊤}\right\}, \end{array}$$

(8) $$\begin{array}{cc} O_{2}\cap O_{3} & =\left\{{\left[\begin{array}{cc} x_{3,1} & y_{3,1} \end{array}\right]}^{⊤},{\left[\begin{array}{cc} x_{3,2} & y_{3,2} \end{array}\right]}^{⊤}\right\}. \end{array}$$

Here, under Assumption 3, the existence of the intersections is proved in the following lemma.

**Lemma 2.**  *Under Assumption 1 and 3, there exist two distinct intersections for each pair $\left(O_{1},O_{2}\right)$, $\left(O_{1},O_{3}\right)$, $\left(O_{2},O_{3}\right)$.*

**Proof.**  It is easy to show that there are two distinct intersections between the two circles $O_{i}$ and $O_{j}$ if $∥{\left[\begin{array}{cc} x_{i} & y_{i} \end{array}\right]}^{⊤}-{\left[\begin{array}{cc} x_{j} & y_{j} \end{array}\right]}^{⊤}∥<2r$ for i,j∈{1,2,3|i≠j}. From Assumption 1, $0\leq d_{i}\leq d$, and $0\leq d_{j}\leq d$, we have

$$\begin{array}{cc} \; & {∥{\left[\begin{array}{cc} x_{i} & y_{i} \end{array}\right]}^{⊤}-{\left[\begin{array}{cc} x_{j} & y_{j} \end{array}\right]}^{⊤}∥}^{2}\\ \; & ={\left(r+d_{i}-d/2\right)}^{2}+{\left(r+d_{j}-d/2\right)}^{2}-2\left(r+d_{i}-d/2\right)\left(r+d_{j}-d/2\right)\cos\left(\theta_{i}-\theta_{j}\right)\\ \; & \leq 2{\left(r+\frac{d}{2}\right)}^{2}-2\left(r+d_{i}-\frac{d}{2}\right)\left(r+d_{j}-\frac{d}{2}\right)\cos\left(\theta_{i}-\theta_{j}\right). \end{array}$$

1. If $90\leq |\theta_{i}-\theta_{j}|\leq 270$ for any i,j∈{1,2,3|i≠j}, it follows from $-1\leq \cos(\theta_{i}-\theta_{j})\leq 0$ and $|\cos\left(\theta_{i}-\theta_{j}\right)|<2{\left(\frac{r}{r+d/2}\right)}^{2}-1$ of Assumption 3 that
  $$\begin{array}{cc} {∥{\left[\begin{array}{cc} x_{i} & y_{i} \end{array}\right]}^{⊤}-{\left[\begin{array}{cc} x_{j} & y_{j} \end{array}\right]}^{⊤}∥}^{2} & \leq 2{\left(r+\frac{d}{2}\right)}^{2}+2{\left(r+\frac{d}{2}\right)}^{2}\left|\cos\left(\theta_{i}-\theta_{j}\right)\right|\\ & <4r^{2}. \end{array}$$
2. Otherwise, it follows from $0\leq \cos(\theta_{i}-\theta_{j})\leq 1$ and $d<2(\sqrt{2}-1)r$ of Assumption 3 that
  $$\begin{array}{cc} {∥{\left[\begin{array}{cc} x_{i} & y_{i} \end{array}\right]}^{⊤}-{\left[\begin{array}{cc} x_{j} & y_{j} \end{array}\right]}^{⊤}∥}^{2} & \leq 2{\left(r+\frac{d}{2}\right)}^{2}\\ & <4r^{2}. \end{array}$$

Therefore, the proof is complete. □

In fact, Assumption 3 required for the proof of Lemma 2 is only sufficient. Thus, failure of Assumption 3 does not mean that two intersection points do not exist. Next, the following lemma shows the relationship between the wafer center and the intersections.

**Lemma 3.**  *The wafer center ${\left[\begin{array}{cc} x_{c} & y_{c} \end{array}\right]}^{⊤}$ is located at one of the two intersections for each pair $\left(O_{1},O_{2}\right)$, $\left(O_{1},O_{3}\right)$, $\left(O_{2},O_{3}\right)$, i.e.,*

$$\begin{array}{c} {\left[\begin{array}{cc} x_{c} & y_{c} \end{array}\right]}^{⊤}\in \left\{{\left[\begin{array}{cc} x_{i,1} & y_{i,1} \end{array}\right]}^{⊤},{\left[\begin{array}{cc} x_{i,2} & y_{i,2} \end{array}\right]}^{⊤}\right\} \end{array}$$

*for i=1,2,3.*

**Proof.**  Since $∥{\left[\begin{array}{cc} x_{c} & y_{c} \end{array}\right]}^{⊤}-{\left[\begin{array}{cc} x_{i} & y_{i} \end{array}\right]}^{⊤}∥=r$, we note that

(9) $$\begin{array}{c} \left[\begin{array}{cc} x_{c} & y_{c} \end{array}\right]\in O_{i} \end{array}$$

for i=1,2,3. Then, it follows from Equation (9) that we have

$$\begin{array}{c} {\left[\begin{array}{cc} x_{c} & y_{c} \end{array}\right]}^{⊤}\in O_{1}\cap O_{2}\cap O_{3}. \end{array}$$

This implies that

$$\begin{array}{c} {\left[\begin{array}{cc} x_{c} & y_{c} \end{array}\right]}^{⊤}\in O_{1}\cap O_{2},\;{\left[\begin{array}{cc} x_{c} & y_{c} \end{array}\right]}^{⊤}\in O_{1}\cap O_{3},\;{\left[\begin{array}{cc} x_{c} & y_{c} \end{array}\right]}^{⊤}\in O_{2}\cap O_{3}. \end{array}$$

Therefore, by the Equations (6)–(8), the proof is complete. □

Finally, it follows from Lemma 3 that we obtain the center of wafer as follows:

(10) $$\begin{array}{c} \left[\begin{array}{c} x_{c}\\ y_{c} \end{array}\right]=\left[\begin{array}{c} x_{1,k}\\ y_{1,k} \end{array}\right] \end{array}$$

where k∈{1,2} such that

(11) $$\begin{array}{c} \left[\begin{array}{c} x_{1,k}\\ y_{1,k} \end{array}\right]\in O_{2}\;\mathrm{and}\;\left[\begin{array}{c} x_{1,k}\\ y_{1,k} \end{array}\right]\in O_{3}. \end{array}$$

Indeed, it is not easy to find *k* that exactly satisfies the Equation (11) because of the sensor measurement error in practice. Thus, instead of (10), we present a method as follows:

$$\begin{array}{cc} x_{c} & =\frac{x_{1,k_{1}}+x_{2,k_{2}}+x_{3,k_{3}}}{3},\\ y_{c} & =\frac{y_{1,k_{1}}+y_{2,k_{2}}+y_{3,k_{3}}}{3} \end{array}$$

where $k_{1},k_{2},k_{3}\in \left\{1,2\right\}$ such that $q(k_{1},k_{2},k_{3})$ has a minimum value among all q(·,·,·), in which, $q(k_{1},k_{2},k_{3})$ is given by

$$\begin{array}{cc} q(k_{1},k_{2},k_{3}) & :=∥\left[\begin{array}{c} x_{1,k_{1}}\\ y_{1,k_{1}} \end{array}\right]-\left[\begin{array}{c} x_{2,k_{2}}\\ y_{2,k_{2}} \end{array}\right]∥+∥\left[\begin{array}{c} x_{1,k_{1}}\\ y_{1,k_{1}} \end{array}\right]-\left[\begin{array}{c} x_{3,k_{3}}\\ y_{3,k_{3}} \end{array}\right]∥+∥\left[\begin{array}{c} x_{2,k_{2}}\\ y_{2,k_{2}} \end{array}\right]-\left[\begin{array}{c} x_{3,k_{3}}\\ y_{3,k_{3}} \end{array}\right]∥. \end{array}$$

### 2.2. Notch Detection Algorithm

As shown in Figure 3, the notch on the wafer edge is one of the major obstacles to obtaining the correct wafer center. To solve the problem, a strategy using rotational motion was proposed in [20]. Instead of the rotational motion, we propose an algorithm with a prismatic motion of Figure 2 to archive the wafer center even if the notch is placed on the sensor. The proposed algorithm proceeds sequentially as follows:

1. Start
2. From the algorithm of Section 2.1, find the intersections and let $v_{1}:={\left[\begin{array}{cc} x_{1,k_{1}} & y_{1,k_{1}} \end{array}\right]}^{⊤}$, $v_{2}:={\left[\begin{array}{cc} x_{2,k_{2}} & y_{2,k_{2}} \end{array}\right]}^{⊤}$, $v_{3}:={\left[\begin{array}{cc} x_{3,k_{3}} & y_{3,k_{3}} \end{array}\right]}^{⊤}$.
3. If $∥v_{1}-v_{2}∥+∥v_{1}-v_{3}∥+∥v_{2}-v_{3}∥$ is less than ϵ>0, jump to 7.
4. Move the robot hand holding the wafer by α in the *X-direction* where α is a positive number.
5. Similar to 2, try again and define ${\bar{v}}_{1}:={\left[\begin{array}{cc} x_{1,k_{1}} & y_{1,k_{1}} \end{array}\right]}^{⊤}$, ${\bar{v}}_{2}:={\left[\begin{array}{cc} x_{2,k_{2}} & y_{2,k_{2}} \end{array}\right]}^{⊤}$, ${\bar{v}}_{3}:={\left[\begin{array}{cc} x_{3,k_{3}} & y_{3,k_{3}} \end{array}\right]}^{⊤}$.
6. Select $v_{j}$ as the center of wafer where *j* is chosen to minimize $|v_{i}^{⊤}\cdot e_{y}-{\bar{v}}_{j}^{⊤}\cdot e_{y}|$ for j=1,2,3 and $e_{y}={\left[\begin{array}{cc} 0 & 1 \end{array}\right]}^{⊤}$. Then, jump to 8.
7. Select $\frac{\left(v_{1}+v_{2}+v_{3}\right)}{3}$ as the center of wafer.
8. End.

Here, ϵ and α are suitable design parameters. Because it is necessary to change the sensor value through the motion in the *X-direction*, it is also supposed that $\theta_{i}\neq 90^{\circ }$ and $\theta_{i}\neq 270^{\circ }$ for i=1,2,3.

## 3. Experimental Results

**Figure 4:**
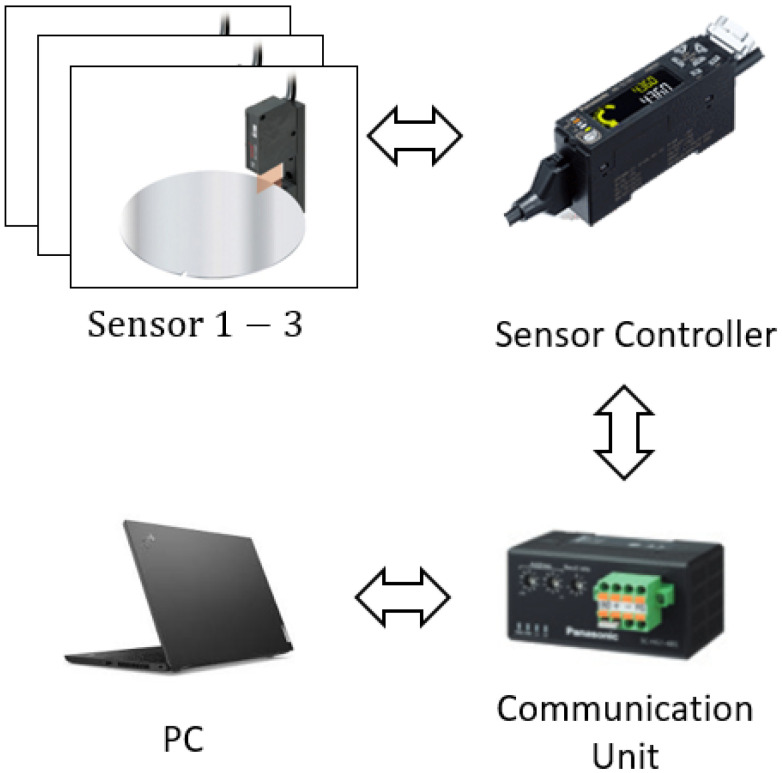
Experimental setup.

Here, the proposed wafer alignment algorithm is implemented for an actual 300 mm coater/developer system in semiconductor processing for verifying the performance of the method as shown in Figure 4. For this, the thru-beam type digital displacement sensors and controllers (HG-T series manufactured by Panasonic Industry Company) are used in the experiment. To transmit the sensor data to the PC, RS-485 communication is used. The measurable sensor range is *d* = 5 mm and the angles of installed sensors are $\theta_{1}=5^{\circ },\theta_{2}=150^{\circ }$, and $\theta_{3}=210^{\circ }$. It is noted that the parameter $r,d,\theta_{1},\theta_{2}$, and $\theta_{3}$ satisfies Assumption 3.

**Figure 5:**
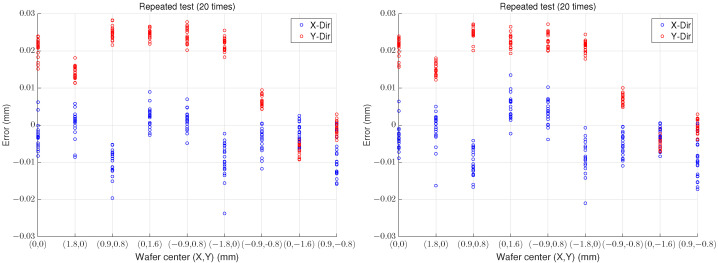
Experimental results of the proposed algorithm (**left**) and the conventional algorithm (**right**).

Figure 5 shows the experimental results of the proposed algorithm and the conventional algorithm of [17], respectively, in which, axis-x and axis-y imply the actual center coordinates of the wafer and the error between it and the calculated center using the proposed algorithm in the paper, respectively. As shown in Table 1, the peak-to-peak error in both algorithms is less than about 0.022 mm in the x and y directions, respectively (The upper bound of the specification is 0.2 mm).

**Figure 6:**
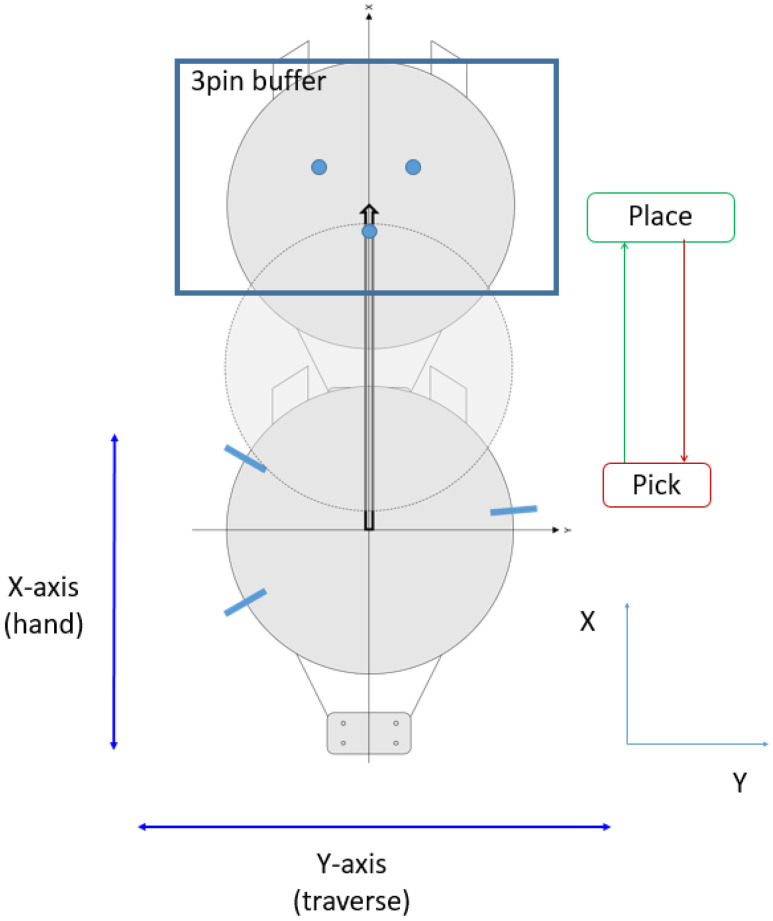
Wafer pick and place for experiments.

As shown in Figure 6, through the pick and place operation, the robot transfers the wafer to the desired position. The *X-axis* is capable of high-precision motion with short strokes, while the *Y-axis* is the traverse direction that moves the robot body, and, thus, the motion precision of the *Y-axis* is relatively low. Therefore, although the required specifications are satisfied, the error in the *Y-direction* is larger than that in the *X-direction*.

To verify the ability of the notch detection algorithm proposed in Section 2.2, we carry out an experiment when the wafer notch is located on the sensor 3. The parameters for the notch algorithm chosen as

$$\begin{array}{c} \epsilon =0.3\;\mathrm{and}\;\alpha =1. \end{array}$$

**Figure 7:**
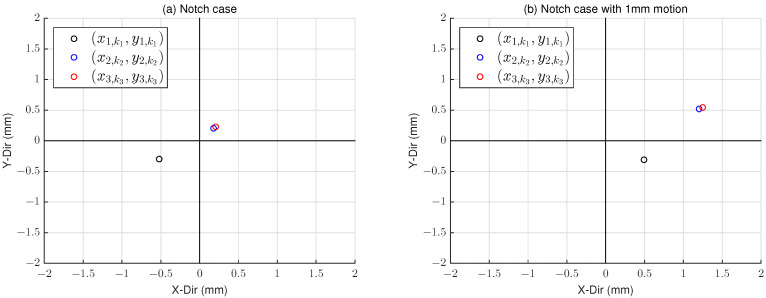
Experimental results of notch case (**a**) and after 1 mm motion in the *X-direction* (**b**).

As shown in Figure 7a, the point ${\left[\begin{array}{cc} x_{1,k_{1}} & y_{1,k_{1}} \end{array}\right]}^{⊤}$ is relatively far from ${\left[\begin{array}{cc} x_{2,k_{2}} & y_{2,k_{2}} \end{array}\right]}^{⊤}$ and ${\left[\begin{array}{cc} x_{3,k_{3}} & y_{3,k_{3}} \end{array}\right]}^{⊤}$, i.e., $q(k_{1},k_{2},k_{3})>\epsilon$. To solve the problem, by the notch detection algorithm, the wafer is moved by α = 1 mm in the *X-direction*. Then, the point ${\left[\begin{array}{cc} x_{1,k_{1}} & y_{1,k_{1}} \end{array}\right]}^{⊤}$ only moves in the *X-direction*, but the rest points also have the *Y-direction* movement, as shown in Figure 7b. Thus, we can select ${\left[\begin{array}{cc} x_{1,k_{1}} & y_{1,k_{1}} \end{array}\right]}^{⊤}$ as the wafer center (see the notch detection algorithm in Section 2.2).

## 4. Conclusions

In this brief, a wafer alignment algorithm has been presented for obtaining the relative distance between the wafer center and the robot hand center. The proposed algorithm can have been designed with only three sensor data less than the conventional algorithm, and, therefore, it has achieved low cost and less computing power. It has been verified that its performance is similar to the conventional method through a actual robot system consisting of three thru-beam type digital displacement sensors.

## Figures and Tables

**Table 1:** Peak-to-peak error for the 20 times repeated test (unit: mm).

Wafer Center X, Y	Proposed		Conventional	
	X-Dir	Y-Dir	X-Dir	Y-Dir
(0,0)	0.0145	0.0087	0.0153	0.0083
(1.8,0)	0.0143	0.0068	0.0213	0.0059
(0.9,0.8)	0.0144	0.0067	0.0125	0.0071
(0,1.6)	0.0116	0.0047	0.0157	0.0072
(−0.9,0.8)	0.0119	0.0076	0.0141	0.0072
(−1.8,0)	0.0215	0.0071	0.0202	0.0066
(−0.9,−0.8)	0.0122	0.0051	0.0106	0.0052
(0,−1.6)	0.0088	0.0067	0.0089	0.0062
(0.9,−0.8)	0.0162	0.0070	0.0177	0.0068

## Data Availability

Not applicable.
